# Activated platelet rich plasma modulates the proliferation, apoptosis and matrix synthesis driven by growth factors in osteoarthritic chondrocytes: an *in vitro* study

**DOI:** 10.3389/fbioe.2026.1940844

**Published:** 2026-09-15

**Authors:** Özge Boyacıoğlu, Bilge Başak Fidan, Mustafa Çelebier, Petek Korkusuz, Feza Korkusuz

**Affiliations:** 1 Department of Medical Biochemistry, Faculty of Medicine, Atılım University, Ankara, Türkiye; 2 Department of Bioengineering, Graduate School of Science and Engineering, Hacettepe University, Ankara, Türkiye; 3 Department of Analytical Chemistry, Faculty of Pharmacy, Hacettepe University, Ankara, Türkiye; 4 Department of Histology and Embryology, Faculty of Medicine, Hacettepe University, Ankara, Türkiye; 5 Metu Mems Center, Ankara, Türkiye; 6 Department of Sports Medicine, Faculty of Medicine, Hacettepe University, Ankara, Türkiye

**Keywords:** apoptosis, autophagy, chondrocyte, osteoarthritis, platelet-rich growth factor, platelet-rich plasma

## Abstract

**Introduction:**

Activated platelet‐rich plasma (aPRP) reduces inflammation and promotes regeneration in osteoarthritic knee joints. It may exert therapeutic effects on osteoarthritic chondrocytes (OACs) compared with healthy chondrocytes (HCs) by promoting cell proliferation, reducing autophagy‐related vesicles and inhibiting apoptosis through modulation of intracellular signaling pathways. The aim of this study was therefore to evaluate cell viability, apoptotic fate, extracellular matrix protein expression (collagen type II (COL2A1) and aggrecan (ACAN)), autophagy‐related vesicle production, and growth factor profiles, including TGF‐β and PDGF-BB, in HCs and OACs.

**Methods:**

Mechanically aPRP‐S and PRP‐G using mini-beads, leukocyte-rich L‐PRP, leukocyte-poor P‐PRP and platelet-rich in growth factors (PRGF) were co-cultured with HCs and OACs for real-time proliferation analysis, apoptosis by flow cytometry, autophagy and related intracellular markers through immunofluorescent labeling, and the secretome profile by ELISA, aligned with their effective dose (ED50) and optimal ED50 time point.

**Results:**

aPRPs at 2.7% in 2 h and PRGF at 1.8% in 3 h induced proliferation of OACs. PRGF reduced the apoptotic cell ratios of OACs. aPRPs and PRGF promoted COL2A1 and ACAN protein synthesis.

**Discussion:**

The induction of proliferation of the OACs is a promising result that can be translated to clinical applications. aPRPs may have a stimulatory role in OACs. This role can be dose and preparation protocol dependent. aPRPs and PRGF can be preferred to stimulate COL2A1 synthesis and reduce apoptotic cell ratios in cultured OACs.

## 1 Introduction

Osteoarthritis (OA) is the most common form of arthritis, affecting around 500 million people worldwide (Tang et al., 2025). The disease progresses through cycles of inflammation, degeneration, and repair in the articular cartilage, subchondral bone, and surrounding tissues, particularly in conditions that overload the joints, such as familial multiple genetic predisposition (Boer et al., 2021) or high body mass index (Hunt et al., 2020). Nearly half of all individuals may be diagnosed with knee OA at some point in their lives (Tang et al., 2025), while hip OA generally occurs in older people and can lead to severe disability (Arslan et al., 2022). The risk of developing OA continues to increase due to rising rates of overweightness and obesity, which exert stress on joints, promote cellular senescence, and trigger systemic inflammation (Park et al., 2023). First-line non-surgical management comprises core conservative measures, including patient education, structured exercise and skeletal muscle strengthening programmes, weight management, and mechanical unloading or realignment of the joint through braces, orthoses or gait-modifying devices, supported pharmacologically by topical and oral non-steroidal anti-inflammatory drugs (NSAIDs), non-opioid analgesics and, for acute inflammatory flares, intra-articular corticosteroid injections (Bannuru et al., 2019; Kolasinski et al., 2020). Disease-modifying approaches, as defined by the ESCEO algorithm, additionally include symptomatic slow-acting drugs for OA (SYSADOAs), such as prescription-grade glucosamine sulfate and chondroitin sulfate, together with intra-articular hyaluronic acid viscosupplementation (Bruyère et al., 2019). Therefore, there is a pressing need for innovative regenerative therapies that can effectively treat OA using advanced regenerative approaches.

Platelet-rich plasma (PRP) is a promising regenerative approach that alleviates symptoms in early and mid-stage knee OA when non-surgical and disease-modifying approaches fail (Glinkowski et al., 2025). PRP therapies can involve several different forms, including Pure Platelet-Rich Plasma (P-PRP), leukocyte-poor PRP (L-PRP), and platelet-rich fibrin (PRF), which differ in concentration and activation techniques. All PRP formulations are known to contain a potent mix of platelet-derived growth factor (PDGF), epidermal growth factor (EGF), insulin-like growth factor I (IGF-I), transforming growth factor beta (TGF-β), and angiogenic growth factors such as fibroblast growth factors (FGF) and vascular endothelial growth factors (VEGF). They have a high capacity for preventing inflammation, promoting tissue regeneration, chondrogenesis and cell proliferation (Everts et al., 2024). The pro-inflammatory cytokines drive matrix degradation in OA, while meniscal dysfunction impair joint stress and accelerate cartilage loss (Mallia et al., 2025; Ozeki et al., 2022; Papalia et al., 2023). Simultaneously, the infrapatellar fat pad (IPFP) and synovium secrete adipokines and inflammatory mediators, assisting chronic low-grade inflammation, promoting chondrocyte catabolism and apoptosis (Mallia et al., 2025; Wang et al., 2024). Since these pathways directly affect chondrocytes, platelet concentrates with distinct inflammatory and growth factor profiles in a chondrocyte model should be evaluated. A previous study (Fidan et al., 2025) revealed that all PRP formulations exhibit distinct metabolic differences, particularly in inflammatory signaling, redox homeostasis, steroid metabolism, energy production, and platelet activation, as assessed by metabolomic analysis. The preparation protocol determined the biochemical features of the PRPs. These findings therefore underscore the urgent need for standardization in the preparation of PRP and highlight the need to evaluate their efficacy in an *in vitro* experimental setup to guide clinicians in selecting or developing an indication-specific PRP product.

This study hypothesizes that mechanically activated PRP (aPRP) formulations using mini-beads, once optimized for dose and time, will demonstrate a superior therapeutic effect compared to other PRP formulations on OA chondrocytes (OACs) and healthy chondrocytes (HCs), specifically by modulating cell proliferation, autophagy and apoptosis. The aim of this study was therefore to elucidate cell viability, apoptotic fate, extracellular matrix protein expression (collagen type II (COL2A1) and aggrecan (ACAN)), autophagy-related vesicle formation, and growth factor profiles (TGF-β and PDGF-BB) in HCs and OACs modulated by various aPRP formulations, particularly aPRP-S and PRP-G, L-PRP, P-PRP and Platelet-Rich in Growth Factors (PRGF). The effective dose and optimal ED50 time of the most successful PRP formulations on HCs and OACs were determined by real-time proliferation analysis, apoptosis by flow cytometry (FCM), autophagy and related intracellular markers through immunofluorescence (IF), and the secretome profile using Enzyme-Linked Immunosorbent Assay (ELISA).

## 2 Materials and methods

### 2.1 Design and independent and dependent variables

An *in vitro* prospective controlled cell culture study was designed with an 80% test power and 95% confidence interval (G-Power v3.1). Independent variables included the PRP groups (aPRP-S, aPRP-G, P-PRP, L-PRP and PRGF) assessed in OACs and HCs, time points (2, 3 or 5 h) and PRP dilutions (1.0, 2.5, and 5.0%). Dependent variables measured were proliferation, autophagy, apoptosis and ELISA assays. Hacettepe University Non-Invasive Clinical Research Ethics Committee (20 September 2022 and #2022/14-57) approved this study. Informed consent was obtained from all individual participants included in the study.

Prior to PRP treatment, cells were thoroughly washed with PBS to remove residual media. PRP formulations were freshly prepared in medium supplemented with 0.1% FBS and applied to the wells. Conditioned media or cells were subsequently harvested at the indicated time points and processed according to the specified method.

### 2.2 Sample collection and PRP preparation

Six healthy, non-smoking male participants aged 18–24 years (mean age 22.0 ± 1.7 years) with a body mass index (BMI) of 26.0 ± 5.0 kg/m^2^ volunteered for the study. Volunteers not taking medication were included. A volunteer receiving chemotherapy was excluded from the study and replaced with another volunteer due to his abnormal blood cell count. Healthy male volunteers within this narrow age range were intentionally selected to minimize biological variability that could confound the metabolomic comparison of different PRP preparation protocols. Age-related metabolic alterations, chronic diseases, medication use, and sex-specific hormonal fluctuations, particularly those associated with the menstrual cycle, are known to influence circulating metabolites, platelet biology, and plasma composition (Costanzo et al., 2022; Krumsiek et al., 2015).

PRP was prepared using five commercially available kits (aPRP-S, aPRP-G, P-PRP, L-PRP and PRGF) according to the manufacturer’s instructions and as previously published (Fidan et al., 2025). Briefly, PRP-S and PRP-G were prepared using the Quantix IDRIA S+ and Quantix IDRIA G commercial systems, respectively, which mechanically activate platelets through proprietary tube designs incorporating glass beads or gel-based activation systems. L-PRP and P-PRP were prepared using the Neogenesis PRP system with sodium citrate as an anticoagulant and were used as conventional non-aPRP formulations without any additional activation procedure. PRGF was prepared by transferring platelet-poor plasma obtained after centrifugation into a calcium gluconate-containing tube, resulting in chemical activation (Fidan et al., 2025). Similar PRP formulations were characterized and their metabolomic profiles were comprehensively evaluated as previously published (Fidan et al., 2025). Since an appropriate optimization and baseline analytical characterization of these kit-based preparations were thoroughly established (Fidan et al., 2025), the isolation methodology and kit parameters were maintained identically to ensure technical consistency and extensive re-characterization was not duplicated for the new set of PRP formulations.

### 2.3 Cell culture

Human OA (#402OA-05a) and healthy (#402-05a) chondrocytes (both obtained from Cell Applications, United States) were cultured in DMEM F12 basal medium supplemented with 15% fetal bovine serum (FBS), 1% L-glutamine and 1% penicillin-streptomycin in an incubator at 37 °C and 5% CO_2_. Cells were passaged up to 11 times to ensure a homogeneous cell supply for subsequent parallel, high-throughput functional assays.

### 2.4 Real-time proliferation analysis

OACs and HCs that reached 70%–80% confluency were washed with the Hanks' Balanced Salt Solution (HBSS), followed by trypsinization and centrifugation. Cells were seeded into 96-well cell plates coated with gold electrodes at a density of 3000 cells/cm^2^. Real-time proliferation analysis was conducted as previously described (Boyacıoğlu et al., 2021). Once cell indices of both the OACs and HCs reached 1.0, aPRP, L-PRP, P-PRP, and PRGF were applied at dilutions of 1.0%, 2.5%, and 5.0% in complete medium. To preserve physiological signaling and reduce excessive cytotoxicity, we optimized the PRP formulations by lowering dose ranges following preliminary optimization based on standard literature references (Chellini et al., 2018; Steller et al., 2019; Amrichová et al., 2014; Jeyakumar et al., 2017), using 10%, 20%, and 50% PRP formulations. Cells treated with medium containing 15% or 0.1% FBS served as control groups. The control conditions, as 15% FBS (positive control) and 0.1% FBS (starved control), were selected, since 15% FBS condition provides an optimal nutrient environment representing the basal proliferation for primary chondrocytes. On the other hand, 0.1% FBS was utilized for serum starvation to eliminate background interference from exogenous cytokines during functional assays. The proliferative efficacy and ED50 of PRPs were determined using the xCELLigence (ACEA, Roche Applied System, Switzerland) device (n = 3). The cell indices were normalized at nearly 1 h of cell seeding, since the cell index reached 1.0 within 1 h. Because the assay specifically targeted the immediate proliferative index, the experimental duration was restricted to 24–26 h, terminating when the cell index in the control and treatment groups returned towards or below 1.0. The ED50 values were calculated within the peak biological activity window, which was determined as hours 4-10, using a sigmoidal dose-response (variable slope) model based on the area under the curve (AUC).

### 2.5 Autophagy assay

The autophagy detection kit (ab139484, Abcam, Cambridge, United Kingdom) was used to assess the autophagy-inducing effect of aPRP and PRGF on OACs and HCs. OACs and HCs were seeded at 2.5x10^4^ cells/well in 24-well culture plates and then assessed for ED50 after activation with aPRP and PRGF. Positive control OACs and HCs were treated with the autophagy inducer rapamycin (500 nM, ab139484, Abcam) and lysosome inhibitor chloroquine (10 μM, ab139484, Abcam) for 16–18 h prior to immunolabeling. The negative control cells were treated only with the rapamycin solvent, DMSO (0.1% v/v) (ab139484). Following the washing step with 1X Assay Buffer, an autophagosome labeling agent (Microscopy dual detection reagent) was applied to cells. The cells were then incubated for 30 min at 37 °C in a 5% CO_2_ incubator, protected from light. Subsequently, cells were washed with 100 µL of assay buffer, fixed in 4% formaldehyde solution, and then washed three times. Cells were visualized under an inverted fluorescence microscope (Olympus IX73, Tokyo, Japan) at excitation and emission wavelengths of 360 nm and 520 nm, respectively. Four different fields were imaged in each well and the number of autophagic vacuole-labeled cells was calculated as a ratio to the total number of cells in the field (n = 3, N = 12).

### 2.6 Apoptosis assay

Early and late apoptotic and necrotic cells were evaluated using Annexin-V/Propidium Iodide (#640914, Biolegend, California, United States) labeling. OACs and HCs were seeded into 24-well plates at 1.5x10^4^ cells per well, followed by the application of aPRP and PRGF at their ED50. Cells were washed with PBS and trypsinized after incubation. They were centrifuged at 1,200 rpm for 5 min and washed twice. After adjusting the cell concentration to 10^6^/mL with 1X Binding Buffer, the cells were transferred to 12 × 75 mm Falcon tubes. 5 μL of Annexin V and 5 μL of PI were added to the cells, which were then incubated for 15 min at room temperature in the dark. Subsequently, 400 μL of 1X Binding Buffer was added, and samples were analyzed by flow cytometry (Novocyte 2000R Flow Cytometer System, Agilent, United States) within 1 h. The percentages of necrotic, early apoptotic, and late apoptotic cells were assessed (n = 3).

### 2.7 Analysis of ECM production

Cell proliferation and ECM synthesis markers including ACAN and COL2A1 in OACs and HCs were evaluated by immunofluorescence after being treated with aPRP and PRGF at ED50. Cells were washed with PBS and fixed with 4% paraformaldehyde for 30 min. They were then washed three times with PBS to remove paraformaldehyde. 0.1% Triton X-100 was applied to the cells for 15 min to ensure antibody penetration. Cells were treated with 2% bovine serum albumin (BSA) for an hour to block them. They were then incubated overnight with primary antibodies to collagen type II (COL2A1, #M00517-1, Boster Bio, California, United States) or aggrecan (ACAN, #MA3-16888, Thermo Fisher, Massachusetts, United States) in a 0.1% BSA solution. After overnight incubation, cells were washed three times with PBS and incubated with rabbit anti-mouse IgG Alexa Fluor 488 (A11059, Life Technologies, California, United States) or goat anti-mouse IgG Alexa Fluor 546 (A11003, Invitrogen, Massachusetts, United States) secondary antibodies for 45 min. After washing three times with PBS, the cells were treated with 100 ng/mL DAPI nuclear stain for 5 min and washed twice. Immunolabeling was visualized under an inverted fluorescence microscope (Olympus IX73). Five fields were imaged in each well, and the number of ACAN and COL2A1 labeled cells in the field was calculated as a ratio to the total number of cells in the field (n = 3, N = 15).

### 2.8 Cartilage regeneration analysis

TGF-β1 (#E-EL-H1657, Elabscience, Wuhan, China) and PDGF-BB (#E-EL-H1577, Elabscience, Wuhan, China) levels in the cell supernatant of OACs and HCs were evaluated by ELISA. OACs and HCs were seeded in 6-well plates at 2-3x10^4^ cells/well followed by aPRP, PRGF and L-PRP applications at ED50. Since L-PRP provides valuable growth factor yield, it was included in the protein characterization to assess the impact of leukocyte presence on TGF-β and PDGF-BB profiles. Supernatants were collected and processed according to the manufacturer’s protocol. Standard solutions were prepared at room temperature. They were then placed in 96-well plates at 100 μL per well and incubated at 37^o^C for 90 min. Following incubation, all supernatants were withdrawn from the wells. 100 μL of biotinylated detection antibody solution was added per well, and the samples were incubated for an additional 1 h at 37^o^C. All samples were then withdrawn from the wells, followed by three washes with PBS. 100 μL of HRP conjugate was then applied per well and the samples were incubated for 15 min at 37^o^C. Following incubation, the plates were treated with Stop solution. The color change observed depending on the protein concentration was measured by a microplate reader (VersaMax Microplate Reader, Molecular Device, California, United States) at a wavelength of 450 nm (n = 3).

### 2.9 Statistical analysis

The normality of the data distribution was assessed using the Shapiro-Wilk test. All parametric data were analyzed using SPSS 25.0. Pairwise comparisons between two independent cell types (HCs and OACs) under baseline or aPRP or PRGF treatment conditions were conducted using two-tailed independent samples Student’s t-test. Cell-fate distribution (live vs. early/late apoptotic cell ratios), COL2A1/ACAN immunolabeling or ELISA-based protein expression within the same experimental group, and for comparisons of multiple treatment formulations (untreated controls vs. aPRP vs. PRGF) within each cell, one-way analysis of variance (ANOVA) was performed. Post-hoc multiple comparisons between individual treatment groups and their respective untreated controls were carried out using Tukey’s HSD test. GraphPad Prism 8 was used to create the final graphs. Statistical significance was set at a 95% confidence interval (p < 0.05). The results are presented as descriptive statistics.

## 3 Results

### 3.1 aPRP, L-PRP and PRGF induced proliferation in OACs at lower doses and in a shorter time compared to HCs

HCs (Figure 1A) and OACs (Figure 1B) exhibit a fusiform, spindle-shaped morphology with high adherence to culture dishes and a high proliferative capacity. Within the first 10 h, aPRP-S, PRGF and L-PRP increased the proliferation of HCs and OACs compared to their internal controls (Figures 1C,D). aPRP-G and P-PRP did not present an exceptional cell proliferation (Figures 1C,D). The ED50 and effective time-point of aPRP-S were calculated as 2.7% and 5 h (*R* ^2^ = 1) in HCs (Figures 1E,G) and 2.2% and 2 h (*R* ^2^ = 1) in OACs (Figures 1F,H). The ED50 and effective time-point of PRGF in HCs were 1.8% and 3 h (*R* ^2^ = 0.99) (Figures 1I,K). It was 1.6% and 3 h (*R* ^2^ = 0.97) in OACs (Figures 1J,L). The ED50 and effective time-point of L-PRP were calculated as 4.3% and 4 h (*R* ^2^ = 0.98) in HCs (Figures 1M,O) and 3.0% and 3 h (*R* ^2^ = 1) for OACs (Figures 1N,P). aPRP and PRGF were selected for downstream functional assays including apoptosis, extracellular matrix component synthesis and autophagy, since these PRPs exhibited better performance within a tight therapeutic window on OACs and HCs compared to other PRPs including L-PRP. L-PRP was selected to analyze its efficacy solely in the cartilage regeneration analysis by ELISA to assess potential leukocyte-mediated effects on growth factor profiles.

**FIGURE 1:**
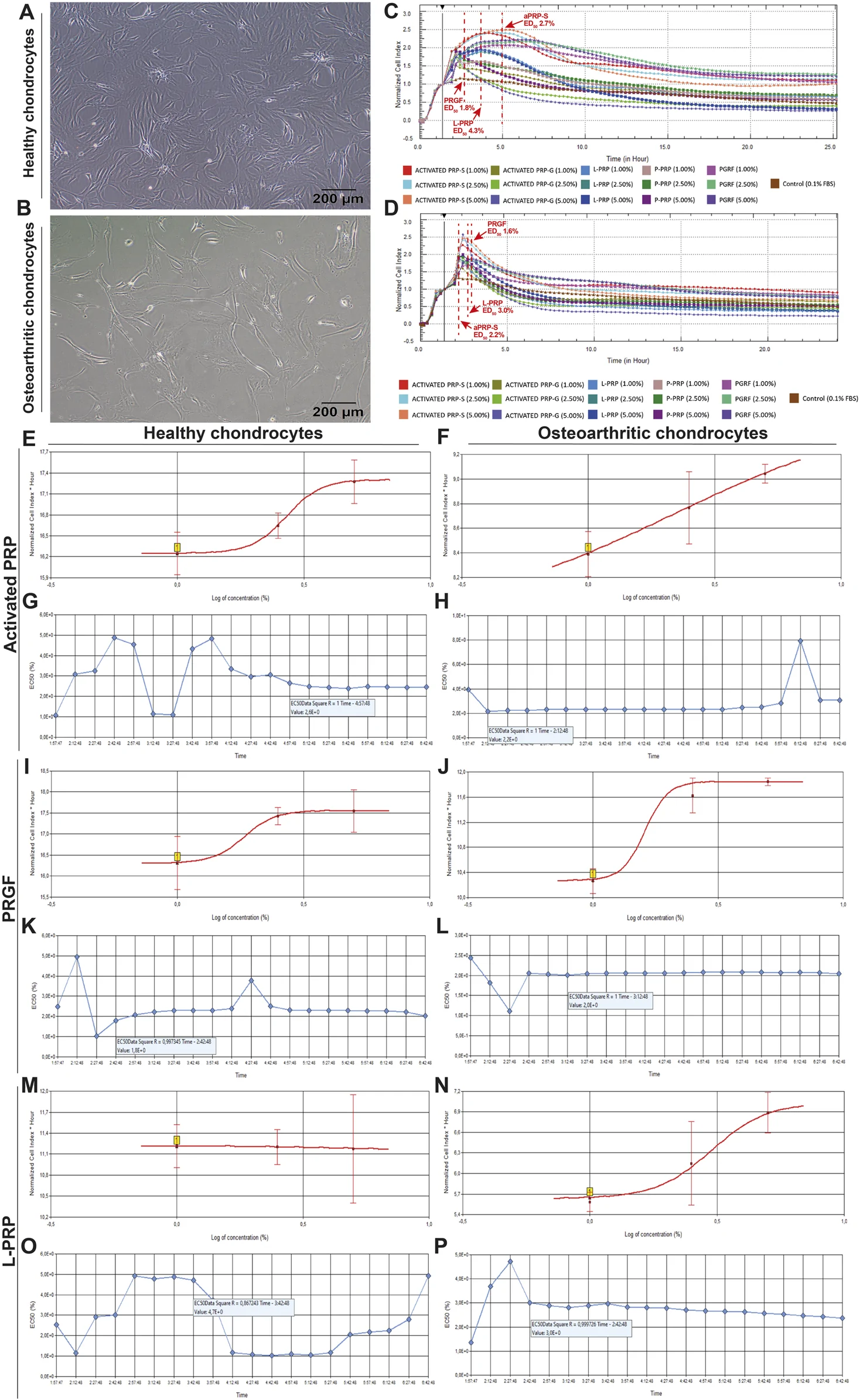
Micrographs of **(A)** HCs and **(B)** OACs with spindle-shaped structures attached to the culture plate are examined. Scale bar 200 μm, 100x. Time-dependent normalized cell index graphs for the real-time and impedance-based measurement of the proliferative effect of aPRP-S, aPRP-G, L-PRP, P-PRP and PRGF on **(C)** HCs and **(D)** OACs, **(E,F,I,J,M,N)** logarithmic concentration vs. normalized cell index*time graphs, and **(G,H,K,L,O,P)** ED50 (%) graphs vs. time are depicted. Vertical dashed lines **(C,D)** indicate the key effective dose time points. ED50 values **(G,H,K,L,O,P)** were derived from Area Under Curve (AUC) modeling using sigmoidal dose-response kinetics within the therapeutic window. Cell indices were normalized nearly at hour 1 and monitored until the proliferative index approached baseline at hours 24-26.

### 3.2 PRGF-applied OACs promoted autophagy-related vacuoles, while aPRP caused similar vacuole formation in both HCs and OACs

HCs and OACs exposed to 2.7% aPRP for 2 h or 1.8% PRGF for 3 h presented autophagy-related vesicles with varying densities and scattered throughout the cytoplasm (Figure 2A). Control OACs exhibited significantly higher extent of immunolabeled autophagy-associated vesicles compared to HCs (Figures 2D,E P = 0.030 for aPRP controls; p = 0.032 for PRGF controls). aPRP (p = 0.890) induced similar autophagy-related vacuole formation in HCs and OACs (Figure 2D), while PRGF-treated OACs displayed significantly elevated autophagy-related vacuole labeling compared to PRGF-treated HCs (p = 0.001). PRGF also induced autophagy-associated vesicle formation compared to aPRP (p = 0.016).

**FIGURE 2:**
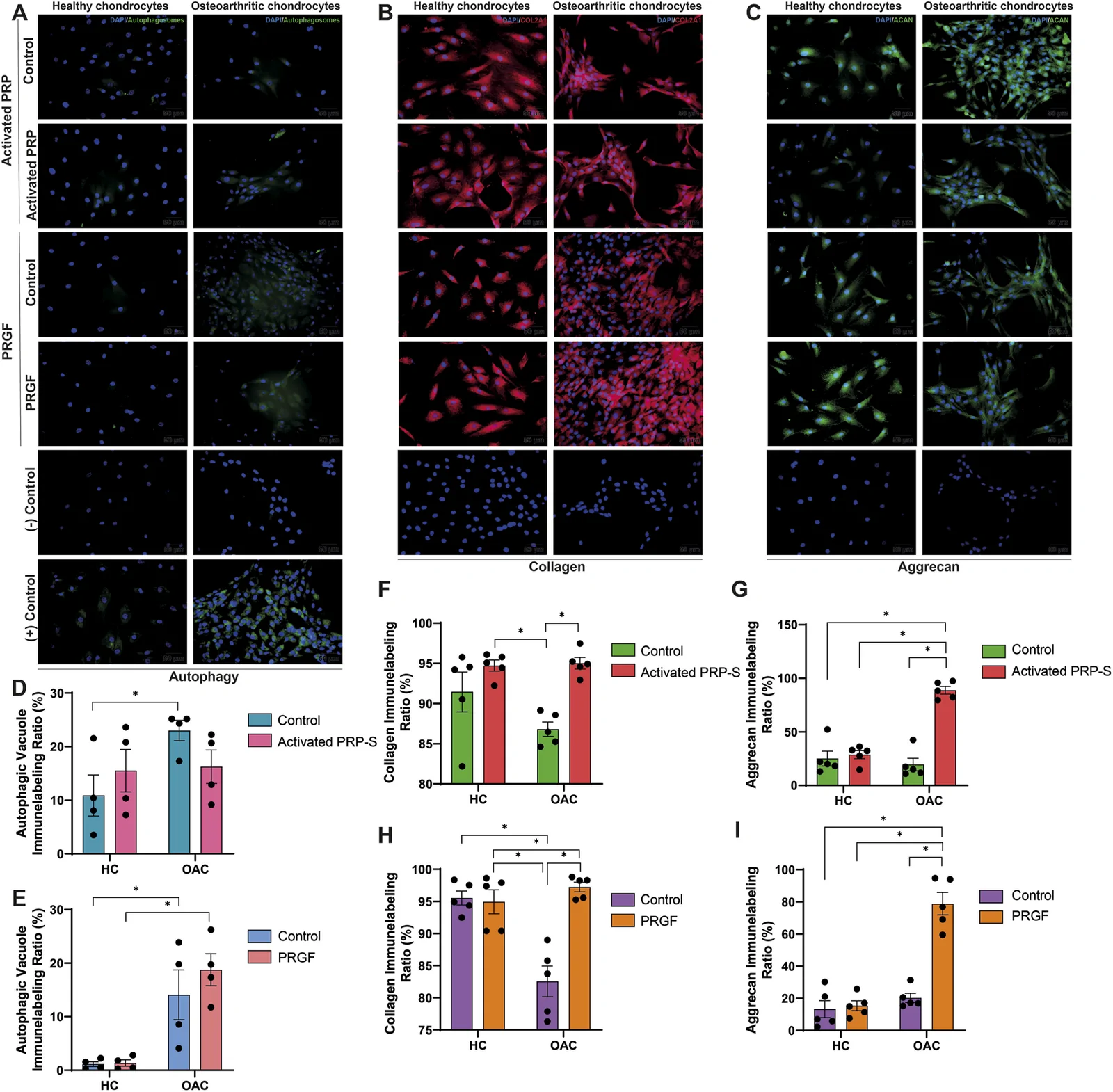
Immunofluorescence labeling of autophagy-associated vacuoles, COL2A1, and ACAN in HCs and OACs. **(A)** FITC-labeled (green) cytoplasmic autophagic vesicle accumulation and Hoechst 33342-labeled (blue) nuclei and **(B)** Alexa Fluor 546-labeled (red) COL2A1 and **(C)** Alexa Fluor 488-labeled (green) ACAN and DAPI-labeled (blue) nuclei are observed following aPRP or PRGF application. Scale bar 50 μm, 400x. Immune labeling ratio (%) of **(D,E)** autophagy-associated vacuoles, **(F–I)** COL2A1 **(F,H)**, and ACAN **(G,I)** in HCs and OACs following aPRP or PRGF application are shown in bar graphs. Pairwise comparisons between two independent cell types (HCs and OACs) by two-tailed independent samples Student’s t-test; Multiple comparisons by one-way analysis of variance (ANOVA), Tukey’s HSD *post hoc* test, (*): p < 0.05.

### 3.3 aPRP and PRGF promoted COL2A1 and ACAN immunolabelling in OACs compared to HCs and controls

Diffuse COL2A1 (Figure 2B) and ACAN (Figure 2C) labeling was observed in the cytoplasm of the 2.7% of aPRP or 1.8% of the PRGF-applied HCs and OACs at 2 or 3 h, respectively. aPRP and PRGF presented elevated COL2A1 labeling in the OACs compared with controls (Figures 2F,H P < 0.05). Specifically, both aPRP (p = 0.003) and PRGF (p < 0.001) significantly increased COL2A1 expression in OACs compared to their untreated controls. Inter-cellular t-test comparisons showed that control OACs exhibited a lower COL2A1 immunolabeling ratio than control HCs (Figure 2H, p = 0.001). aPRP (p = 0.780) or PRGF (p = 0.287) presented similar COL2A1 levels in OACs compared to those of treated HCs.

aPRP and PRGF increased the ACAN immunolabeling ratio in the OACs compared to the control OACs (p < 0.001 for both aPRP and PRGF) and the aPRP-treated HCs (Figures 2G,I). Independent samples t-test revealed significantly higher ACAN induction in OACs compared to HCs following either aPRP (p < 0.001) or PRGF (p < 0.001) treatment.

### 3.4 aPRP promoted apoptotic cell ratio in OACs, while PRGF diminished the ratio in OACs

The ratio of early and late apoptotic cells in aPRP-treated OACs at a dose of 2.7% for 2 h rose from 60% to 80% compared to HCs (Figures 3A–C). aPRP increased the proportion of late apoptotic cells in the OACs relative to HCs and controls (Figures 3A–C). Untreated OAC controls exhibited significantly higher early apoptosis (p = 0.006), late apoptosis (p = 0.001), dead/necrotic cell ratio (p = 0.024), and lower live cell ratio (p < 0.001) compared to HC controls. Following aPRP treatment, OACs maintained higher early apoptosis (p = 0.048), late apoptosis (p = 0.002), and lower live cell ratio (p = 0.001) relative to HCs, whereas dead cell ratios showed no significant difference (p > 0.05). Additionally, 1.8% of PRGF-treated OACs showed a reduced early apoptotic cell ratio (30%) and induced late apoptosis (10%) after 3 h compared to the control and HCs (Figures 3B,D). Inter-cellular Independent Samples T-test comparison also confirmed higher early apoptosis (p < 0.001), dead cells (p = 0.037), and lower live cell ratio (p < 0.001) in OAC controls compared to HC controls, while late apoptosis showed similar trend between HC and OAC controls (p = 0.096). Following PRGF treatment, OACs exhibited significantly higher early apoptosis (p = 0.001), late apoptosis (p = 0.006) and lower live cells (p = 0.008) compared to PRGF-treated HCs, with no difference in dead cells (p > 0.05).

**FIGURE 3:**
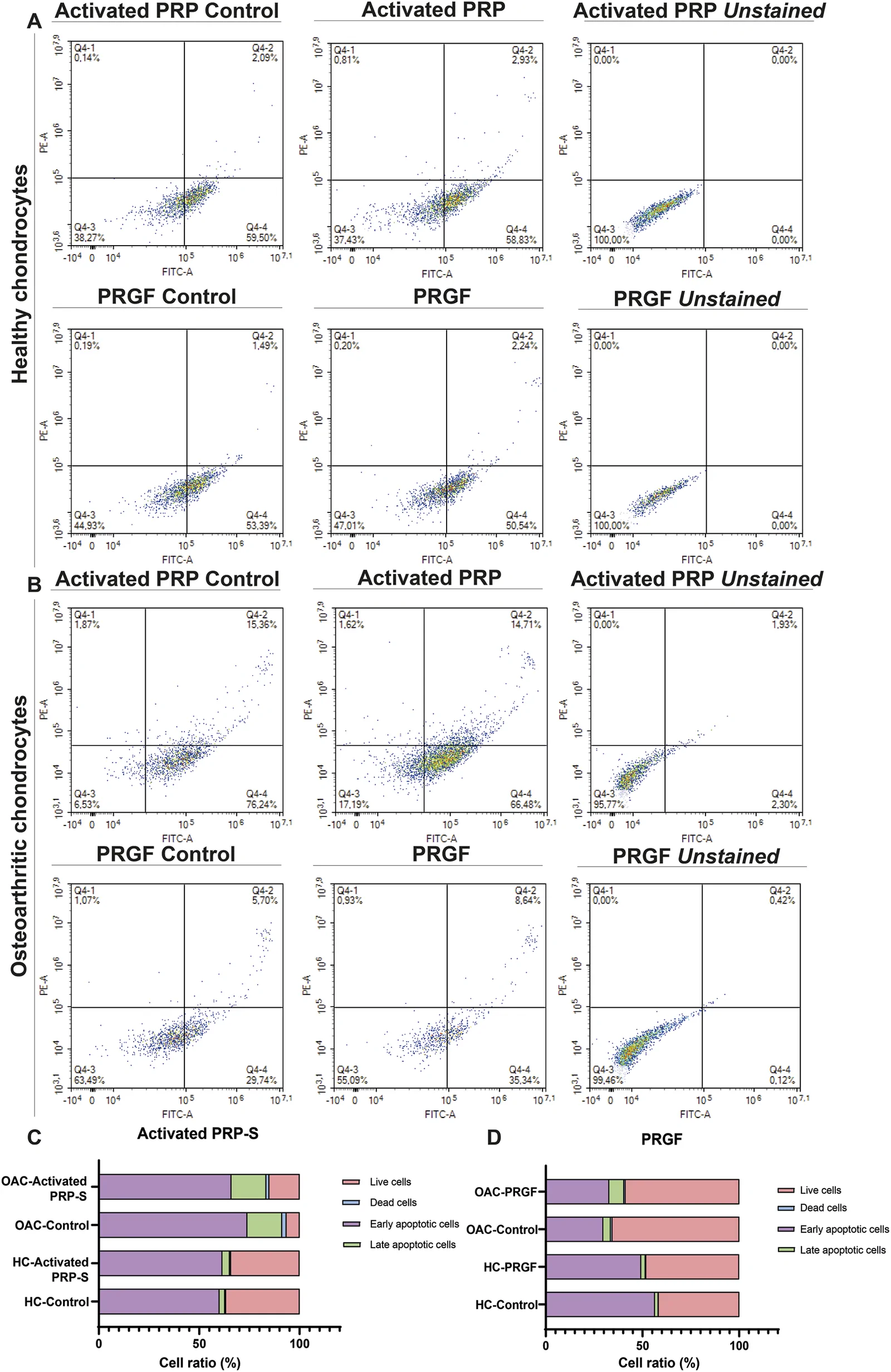
Demonstration of the apoptotic effect of aPRP or PRGF on HCs and OACs at 2 or 3 h by FCM. Early apoptosis, late apoptosis, necrosis, and viability ratio of **(A)** HCs and **(B)** OACs treated with 2.7% aPRP or 1.8% PRGF and **(C,D)** stacked column graphs are depicted. Pairwise comparisons between two independent cell types (HCs and OACs) by two-tailed independent samples Student’s t-test; Multiple comparisons by one-way analysis of variance (ANOVA), Tukey’s HSD *post hoc* test.

Within-group ANOVA and Tukey *post hoc* tests demonstrated that early apoptotic cells significantly exceeded live cells in HC controls (p < 0.001), aPRP-applied HCs (p < 0.001), and OAC controls (p < 0.001; late apoptotic cells vs. live cells p = 0.001). However, in aPRP-treated OACs, late apoptotic and live cell proportions were similar (p = 0.498), while early apoptotic cells were higher than live cells (p < 0.001). PRGF treatment in HCs balanced early apoptotic and live cell ratios to approximately 50:50 (p > 0.05). In OAC controls, early apoptosis exceeded late apoptosis (p < 0.001), late apoptosis was lower than live cells (p < 0.001) and live cells represented the highest ratio compared to late and early apoptotic cell and necrotic/dead cell ratios (p < 0.001). Following PRGF treatment in OACs, early apoptosis remained higher than late apoptosis (p < 0.001), accompanied by a slight increase in late apoptosis relative to its control.

aPRP significantly reduced early apoptosis (p = 0.003) and increased overall live cell ratio (p = 0.009) in OACs without altering late apoptosis (p = 1.000), while exerting no significant changes in HCs (p = 0.719 for live cells; p > 0.05 for early and late apoptotic cells) compared to its control. PRGF did not alter the ratio of live (p = 0.092), early, or late apoptotic cell ratios in HCs (Figures 3D P > 0.05), but significantly reduced OAC viability (p = 0.032) while showing non-significant trends in early (p = 0.226) and late (p = 0.264) apoptotic cell ratios.

### 3.5 aPRP, PRGF and L-PRP elevated TGF-β and PDGF-BB expression in HCs and OACs

2.7% of aPRP-applied HCs and OACs presented higher TGF-β concentrations compared to the controls at 2 and 5 h (Figures 4A,D). Specifically, aPRP increased TGF-β levels in both HCs (p < 0.001) and OACs (p < 0.001) compared to their untreated controls at 2 h, with no significant difference observed between aPRP-treated HCs and OACs (p > 0.05). aPRP maintained elevated TGF-β levels in OACs compared to control (p = 0.012) at 5 h aPRP did not significantly alter PDGF-BB expression in HCs or OACs at 2 h (Figures 4E P > 0.05) and 5 h (Figures 4H P > 0.05).

**FIGURE 4:**
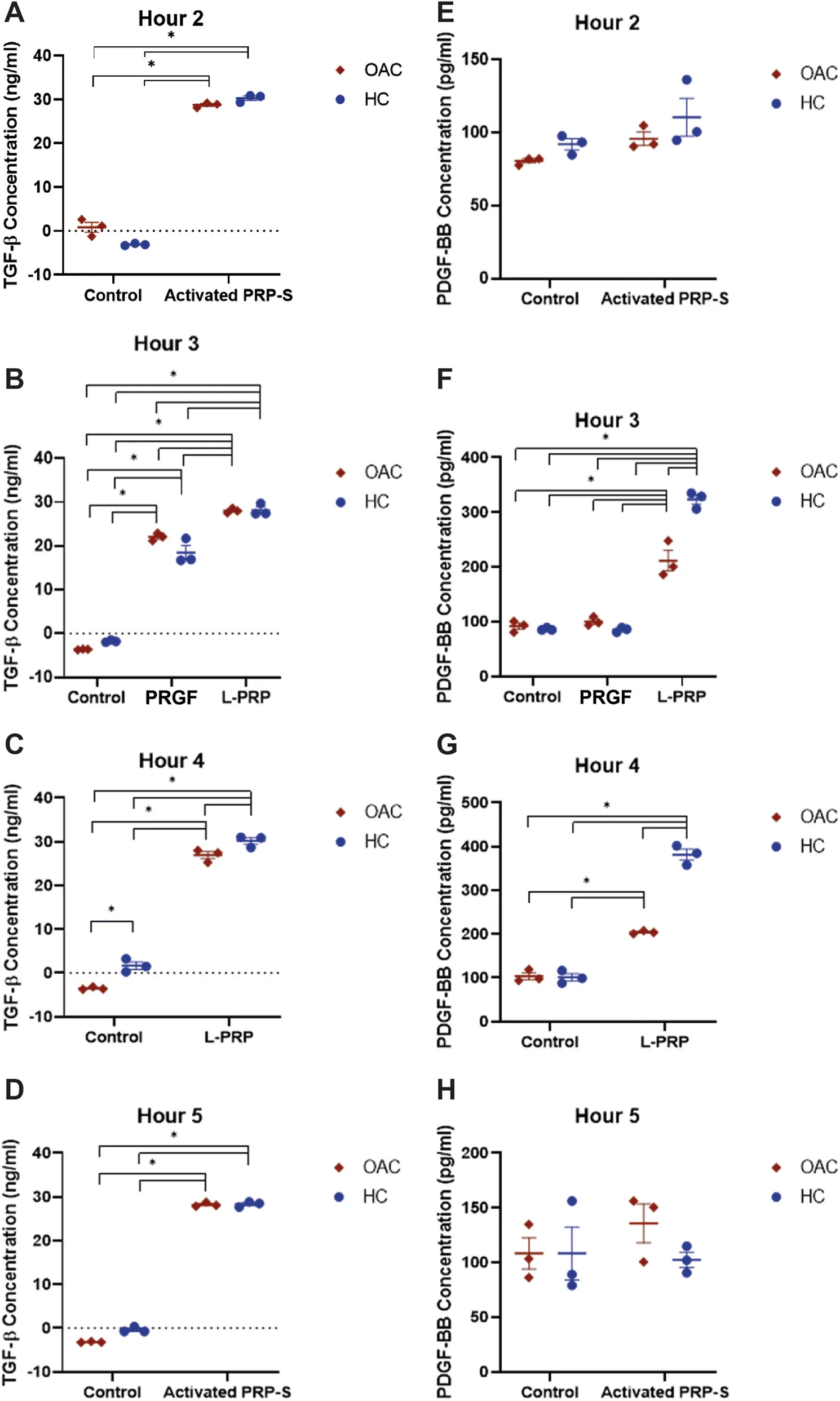
Demonstration of the effect of aPRP or PRGF on TGF-β and PDGF-BB expression in HCs and OACs at 2 or 3 h by ELISA. **(A–D)** TGF-β and **(E–H)** PDGF-BB expressions of 2.7% aPRP, 1.8% PGRF, or 4.3% L-PRP-administered HCs and OACs are given as mean standard deviation graphs for each time point in comparison with the control groups. One-way analysis of variance (ANOVA), Tukey’s HSD *post hoc* test (*): p < 0.05.

PRGF at 1.8% or L-PRP at 4.3% dose elevated TGF-β or PDGF-BB concentrations in HCs and OACs compared to control at 3 and 4 h (Figures 4B,C,G). PRGF and L-PRP increased TGF-β levels in HCs (p < 0.001) and OACs (p < 0.001) compared to their controls at 3 h, while L-PRP induced TGF-β levels similarly in HCs and OACs (p > 0.05). PRGF-treated OACs produced higher TGF-β levels than L-PRP-treated OACs (p = 0.002), while exhibiting similar trend compared to PRGF-treated HCs (p = 0.056). L-PRP boosted TGF-β levels in HCs (p < 0.001) and OACs (p < 0.001) compared to their controls, while L-PRP-treated OACs exhibited higher expression than L-PRP-treated HCs (p = 0.047) at 4 h. TGF-β levels were also higher in control OACs than control HCs at 4 h (p = 0.004).

4.3% of L-PRP-applied HCs exhibited higher PDGF-BB expression at 3 h compared to L-PRP-applied OACs and controls (Figure 4F, for all groups p < 0.001). L-PRP increased PDGF-BB expression in OACs compared to that of its control at 3 h (Figure 4F, p < 0.001). At 4 h, L-PRP maintained significantly elevated PDGF-BB expression in HCs and OACs compared to their controls (p < 0.001), with L-PRP-treated HCs displaying significantly higher PDGF-BB levels than L-PRP-treated OACs (p < 0.001).

## 4 Discussion

In this study, aPRP and PRGF at 2.7% and 1.8%, respectively, induced proliferation in OACs at 2 and 3 h compared to HCs. PRGF at ED50 promoted autophagy-related vacuoles in OACs, whereas aPRP presented similar autophagy-associated vesicle labeling in both HCs and OACs. Both aPRP and PRGF promoted COL2A1 and ACAN protein expression in OACs compared to HCs and control groups. aPRP promoted apoptosis in OACs, while PRGF reduced the apoptotic cell ratios in OACs. aPRP, PRGF and L-PRP elevated TGF-β and PDGF-BB expression in HCs and OACs (Figure 5).

**FIGURE 5:**
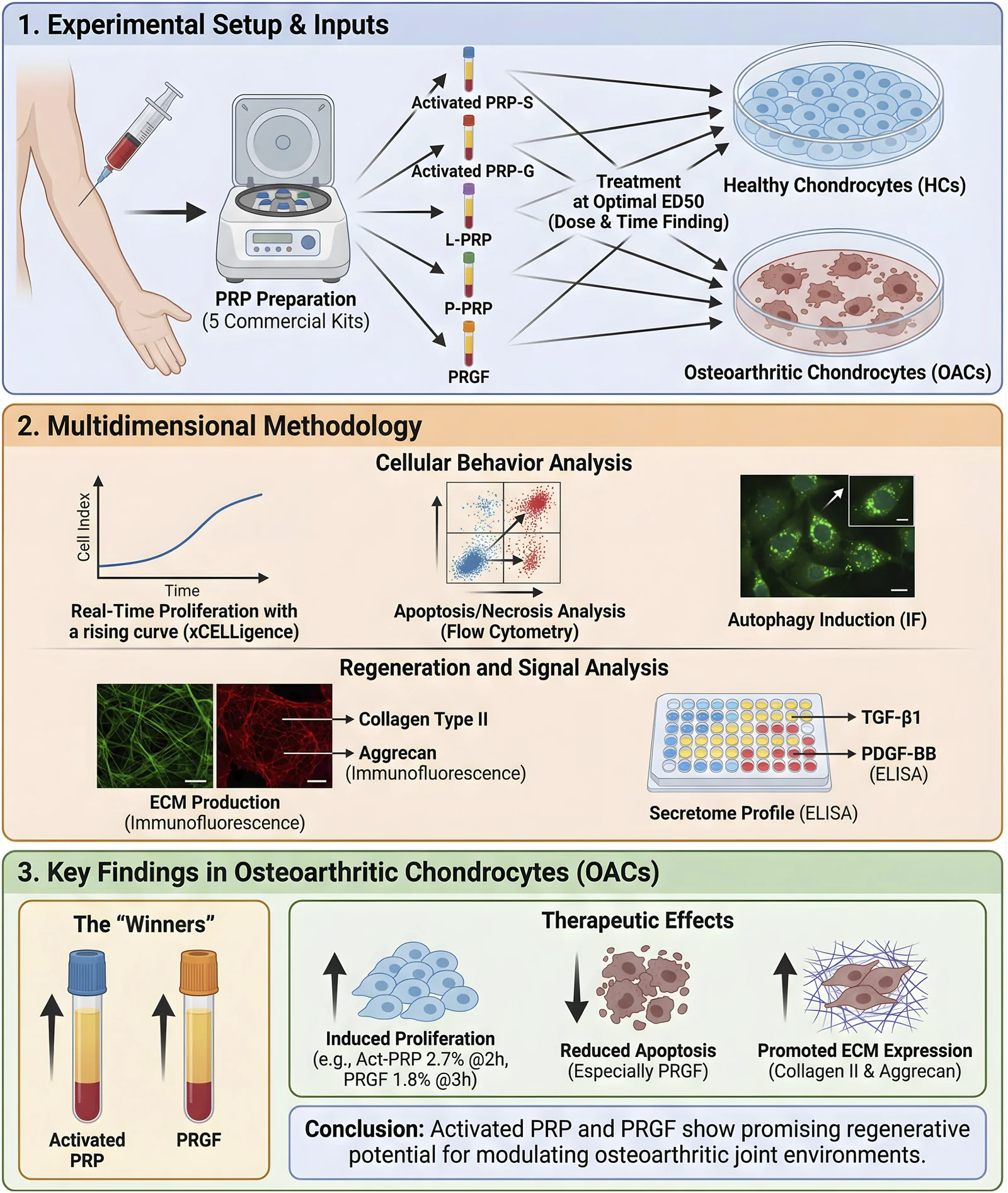
Flow diagram illustrates the overall design and study outcomes. Activated PRP at lower EC50 promotes OAC proliferation and apoptosis and induces collagen type-II, aggrecan, TGF-β and PDGF-BB expressions in OACs. Activated PRP may improve osteoarthritic joint function. This figure was created with AI assistance under the authors’ supervision. All conceptual content and final approval were provided by the authors.

The five formulations examined here differ in leukocyte content, platelet enrichment and activation mode. P-PRP and PRGF are leukocyte-poor preparations, while L-PRP retains the buffy-coat leukocyte fraction. This distinction is biologically pivotal, since P-PRP and L-PRP may contain comparable platelet counts and comparable PDGF-AB, TGF-β1 and VEGF levels while L-PRP carries significantly higher IL-1β and TNF-α, which activate NF-κB signaling and impair cell proliferation, migration and differentiation (Yin et al., 2016). PRGF is activated with calcium chloride, without exogenous thrombin and its leukocyte-free composition yields a minimal pro-inflammatory load (Masuki et al., 2016). The two mechanically activated kits, aPRP-S and aPRP-G, differ from the others in activation principle rather than in cellular content. Mini-bead mediated mechanical activation triggers platelet degranulation and fibrin polymerization without exogenous biochemical activators, so that the growth factor pool is released progressively from the forming fibrin matrix rather than as a burst effect (Masuki et al., 2016; Husak et al., 2025). Accordingly, the five products should be regarded not as different doses of the same agent, but as biochemically distinct formulations whose leukocyte load and release profiles determine their behaviour on HCs and OACs.

The ED50 and activation time point for PRP and PRGF in OACs and HCs were identified through real-time proliferation analysis, demonstrating their peak effectiveness compared to other PRP formulations. aPRP showed an ED50 of 2.7% at 2 h, while PRGF exhibited an ED50 of 1.8% at 3 h on OACs, in this study. When applied at a dose of 10%, PRP promoted the proliferation of HCs compared to control, as seen in real-time proliferation analysis at 48 h (Amrichová et al., 2014). A dose of 5% PRP increased OAC proliferation relative to control between hours 96 and 192, as measured by MTT (Muraglia et al., 2014). PRP at 1%, 5%, 10% and 20% stimulated rabbit chondrocyte proliferation between 24 and 96 h, according to MTT results (Park et al., 2012). 10% of PRP significantly enhanced the proliferation of canine OACs at 25 h compared to controls (Bianchini et al., 2018). The proliferation data from this study aligned with efficacy levels reported in existing literature. Here, PRP applied at 1%–5% to HCs and OACs showed that PRGF (1.8%), L-PRP (4.3%), and especially aPRP (2.7%) induced maximum proliferative effects at low doses, and within a very short timeframe. As a result, aPRP and PRGF, identified as the most effective low-dose formulations, were chosen for further investigation.

The major novel finding of the present study regarding low ED50 and short therapeutic duration of PRP formulations contrasts with traditional *in vitro* models that rely on high doses and prolonged incubation times, such as 24–48 h, which may not accurately reflect the *in vivo* microenvironment. However, in this study, aPRP and PRGF, more prominently induced robust proliferative or cytoprotective responses in both OACs and HCs. From a translational perspective, intra-articularly injected PRP undergoes immediate dilution within the synovial fluid and is subject to rapid clearance through lymphatic and synovial drainage, resulting in a relatively short joint retention time. Our findings demonstrate that effective PRP formulations do not require excessive dosing to exert biological activity. Instead, an early burst effect (lower than 5 h) at physiologically low-dose threshold might be adequate to trigger primary intracellular signaling pathways. This finding might provide strong preclinical validation for clinical protocols that suggest achieving effective intra-articular cartilage repair without requiring high-concentration or high-volume injections.

In this study, we revealed that aPRP and PRGF at ED50 values of 2.7% and 1.8% significantly increased autophagy-related vacuole labeling to 20% in cytoplasm of OACs compared to control and HCs. Moreover, PRGF induced significantly higher autophagy-related vesicle formation in control HCs compared to aPRP and maintained elevated autophagic activity in OACs compared to treated HCs. 10%–20% of PRP dose-dependently increased the mRNA expression of the autophagic markers BECLIN and LC3II in OACs compared to control (Moussa et al., 2017). 10% of L-PRP or P-PRP did not induce autophagy-related Atg5, Beclin1, or LC3 II/LC3 I gene expression or autophagic vacuole formation in HCs at 24 h (Yang et al., 2018). In our study, PRGF induced high autophagy-associated vesicle labeling at lower doses than reported in the limited literature (Yang et al., 2018; Moussa et al., 2017). Within the scope of this study, aPRP (2.7%) and PRGF (1.8%)-applied OACs enhanced COL2A1 and ACAN labeling compared to control and HCs at 2–3 h. The depletion of COL2A1 in control OACs relative to HCs might reflect degenerative cartilage degradation. The robust upregulation of ACAN in OACs may highlight the capacity of these PRP formulations to stimulate proteoglycan synthesis. 10% of L-PRP or P-PRP increased COL2A1 and ACAN mRNA expression in HCs at 24 and 96 h compared to controls (Yang et al., 2018). 10% of PRP significantly increased COL2A1 mRNA expression in OACs at 24 and 72 h (Jeyakumar et al., 2017). In our study, aPRP and PRGF at very low doses, in our study, induced chondrogenic structural protein labeling through high COL2A1 and ACAN labeling compared with previous findings.

Herein, we demonstrated that aPRP induced early and late apoptosis in OACs, increasing the apoptotic cell rate to 20% compared with HCs. OACs presented higher apoptotic stress across baseline controls and HCs. When exposed to aPRP, OACs demonstrated a significant reduction in early apoptosis alongside a marked increase in total cell viability compared to untreated controls. aPRP also shifted the OAC cellular balance, as the ratio of late apoptotic and live cells became statistically equivalent. This statistical outcome might support a selective elimination mechanism driven by the specific growth factors in aPRP. Conversely, PRGF acted as a cytoprotective agent balancing the early apoptotic and live cell proportions in HCs, neutralizing the basal culture stress without inducing cytotoxicity or changing overall apoptotic parameters. PRGF significantly reduced cell viability in OACs, in this study. We also revealed that aPRP increased TGF-β expression at 2 and 5 h in HCs and OACs, with levels comparable to those of control groups. An immediate, early burst of TGF-β level by aPRP within 2 h remained sustained up to 5 h in OACs, explaining its early impact on chondrocyte proliferation. Effective doses of PRGF and L-PRP induced maximal induction of TGF-β and PDGF-BB expression in HCs and OACs at 3 and 4 h, respectively, compared to controls. This pronounced PDGF-BB release by L-PRP might reflect leukocyte-mediated factor release over different time points. On the other hand, PRGF provided a more balanced upregulation of TGF-β at 3 h, aligning with its cytoprotective mechanism of action. Combined with the apoptosis findings, PRGF influences controlled and protective autophagic recovery to support cell survival and matrix synthesis, while aPRP stimulates these metabolic pathways rapidly alongside selective clearance of dead cells.

A single study on this topic has been reported in the literature. 10% of PRP-induced apoptosis in OACs at 48 h by increasing early and late apoptosis by 10% compared to control by FCM (Li et al., 2022). In the same study, PRP induced collagen, PDGF, and TGF-β mRNA and protein expression in OACs, similar to controls (Li et al., 2022). In this study, aPRP induced apoptosis and triggered matrix synthesis and regeneration by increasing TGF-β and PDGF-BB expression at a lower ED50 than previously reported, in a shorter time. Herein, aPRP promoted proliferation and induced apoptosis in OACs, while it maintained cell distribution profile in HCs, demonstrating no cellular toxicity or stress. PRGF administration enhanced cell viability in HCs while reducing the proportion of early apoptotic HCs compared to control. It also exerted a robust anti-apoptotic efficacy in OACs. Our findings pointing in two distinct directions might represent a selective mechanism due to activation of growth factors and thrombin in PRP (Chen et al., 2025; Patel et al., 2023). This activation of PRP further leads to hyperstimulation of metabolic activities in OACs that pushes degenerated or senescent OACs under stress toward apoptotic clearance (Zhang et al., 2026). Conversely, PRGF demonstrated marked anti-apoptotic effects, which may be attributed to its leukocyte-free nature and a more balanced TGF-β and PDGF-BB ratio. TGF-β activates SMAD2/3 signaling and the pro-survival PI3K/Akt and ERK pathways, inhibiting intrinsic apoptotic pathways especially in degenerative cartilage (Du et al., 2023; Thielen et al., 2019). Therefore, more physiologically balanced content of PRGF might exert cytoprotective efficacy across both HCs and OACs, while aPRP might serve as an accelerator of selective clearance of irreversibly damaged OACs via apoptotic pathways or PI3K/Akt/mTOR pathway, leading to overactivation of autophagy-related vacuole formation (Huang et al., 2025), due to high growth factor, cytokine and bioactive molecule content. This might also disrupt the chondrocyte metabolic homeostasis, elevate intracellular reactive oxygen species (ROS) and subsequently shift cells early apoptotic OACs toward a late apoptotic state.

The output of this study is limited to *in vitro* conditions, which requires further functional *ex vivo* and *in vivo* validation studies. This limitation was mitigated by real-time proliferation analysis, which enabled impedance-based monitoring of cells in real time as an alternative to live-cell imaging. Our primary aim was to demonstrate the acute intracellular effects of PRP derivatives during the early phase of cellular recovery; therefore, the *in vitro* design was appropriate and sufficient for capturing these immediate mechanistic responses. In this context, this study still provides valuable insight and contributes to literature despite the inherent constraints of *in vitro* experimentation. The platelet and leukocyte content of PRPs following PRP preparation was not quantified, which was crucial for clinical practice. However, our aim in this study was not to characterize the PRP formulations for clinical standardization, since they were also previously characterized (Fidan et al., 2025), but to determine the ED50 values of total PRPs. Moreover, the ED50 values obtained from real-time proliferation analysis do not affect their biological activities. Therefore, the functional assessment successfully conducted remains valid and relevant for understanding the acute cellular effects of whole PRP samples. The total volume of each PRP sample was limited. Therefore, all PRP samples from all patients were pooled to minimize inter-individual variability. On the other hand, more comprehensive functional analysis of intracellular signaling pathways involved in proliferation and regeneration was not evaluated and constitutes a crucial limitation of this study, which requires further investigations integrating multi-omics approaches. Moreover, the evaluation of proliferation, apoptosis, secretome profile, and key matrix components, herein, provided robust functional mapping of chondrocyte behavior. However, it does not capture the fully map specific intracellular signaling cascades including specific kinase phosphorylation pathways. Therefore, our preliminary findings support cytoprotective and anabolic pathway modulation by PRP formulations, which requires *in vivo* validation and further mechanistic investigation. Our main aim was to demonstrate the ED50 of each PRP sample on both OACs and HCs complemented with TGF-β1 and PDGF-BB secretome analysis for ECM production and ACAN and COL2A1 protein expressions for cartilage regeneration analysis. Thus, these findings are critical in understanding the major regenerative effects of different allogeneic PRP samples when applied at very low doses that provides a new insight into literature. In the present study, FITC-fluorescence-based autophagy assay demonstrated a notable increase in intracellular autophagy-related vacuole labeling in OACs following PRP treatment. While fluorescent labeling and semiquantitative interpretation provide valuable evidence of autophagy-related vesicle accumulation, the actual autophagy induction should be interpreted with complete characterization of functional autophagic flux that requires mechanistic verification, such as evaluating the conversion ratio of LC3-I to LC3-II and monitoring p62 degradation via Western blot analysis. Therefore, our current observations primarily reflect an accumulation of autophagy-associated vesicles rather than verified flux dynamics. Future mechanistic studies incorporating quantitative immunoblotting of key autophagic markers and flux assays are required to conclusively establish the precise stage of autophagy process in this model.

## 5 Conclusion

In summary, the PRP activated at 2.7% over 2 h and PRGF at 1.8% over 3 h promoted OAC proliferation compared to HCs. This *in vitro* data demonstrates that aPRP supports OAC growth. However, apoptotic vacuoles were seen in both cell types after PRP activation. PRGF reduced the number of apoptotic cells in OACs. Both aPRP and PRGF increased COL2A1 and ACAN protein levels in OACs relative to HCs and controls. Furthermore, aPRP, PRGF, and L-PRP elevated TGF-β and PDGF-BB expression in both HCs and OACs. Clinically, although apoptotic vacuoles appeared in chondrocytes after aPRP treatment, the stimulation of OAC proliferation is encouraging. Overall, along with increased TGF-β and PDGF-BB, this study suggests that aPRP could have beneficial effects in osteoarthritic joints.

## Data Availability

The original contributions presented in the study are included in the article/supplementary material, further inquiries can be directed to the corresponding author.
